# Neuroprotective Effects of Proanthocyanidins, Natural Flavonoids Derived From Plants, on Rotenone-Induced Oxidative Stress and Apoptotic Cell Death in Human Neuroblastoma SH-SY5Y Cells

**DOI:** 10.3389/fnins.2018.00369

**Published:** 2018-05-31

**Authors:** Jian Ma, Shan-Shan Gao, Hai-Jie Yang, Mian Wang, Bin-Feng Cheng, Zhi-Wei Feng, Lei Wang

**Affiliations:** ^1^School of Life Sciences and Technology, Xinxiang Medical University, Xinxiang, China; ^2^Henan Collaborative Innovation Center of Molecular Diagnosis and Laboratory Medicine, Xinxiang Medical University, Xinxiang, China; ^3^Disciplinary group of Psychology and Neuroscience, Xinxiang Medical University, Xinxiang, China

**Keywords:** proanthocyanidin, oxidative stress, apoptosis, p38, JNK, ERK

## Abstract

Proanthocyanidins (PA) are natural flavonoids widely present in many vegetables, fruits, nuts and seeds, and especially in grape seed. In the present study, we examined the neuroprotective effects of PA and the underlying molecular mechanism in rotenone model of Parkinson's disease (PD). We found that pretreatment with PA significantly reduced rotenone-induced oxidative stress in human neuroblastoma SH-SY5Y dopaminergic cells. In addition, PA markedly enhanced cell viability against rotenone neurotoxicity and considerably blocked rotenone-induced activation of caspase-9, caspase-3, and cleavage of poly (ADP-ribose) polymerase (PARP), biochemical features of apoptosis. Further study demonstrated that the anti-apoptotic effect of PA was mediated by suppressing p38, JNK, and ERK signaling, and inhibitors of these three signaling pathways reproduced the protective effect of PA separately. In summary, our results demonstrated that PA mitigated rotenone-induced ROS generation and antagonized apoptosis in SH-SY5Y cells by inhibiting p38, JNK, and ERK signaling pathways, and it may provide a new insight of PA in PD therapy.

## Introduction

Parkinson's disease (PD) is a slowly progressive common neurodegenerative movement disorder, with an increased incidence in persons with advanced age (Dauer and Przedborski, 2003). Clinically, PD is characterized by rest tremor, muscle rigidity, bradykinesia, postural instability and freezing (Jomova et al., 2010). PD is mainly caused by the selective loss of dopaminergic neurons within the substantia nigra pars compacta (SNpc) which is a midbrain structure transmitting signals to the striatum (ST) for motor function coordination, and a subsequent deletion of dopamine in ST (Cacabelos, 2017). Apoptosis may play a cardinal role in the degeneration of dopaminergic neurons in the SNpc, which were supported by the presence of DNA fragments as revealed by terminal deoxynulceotidyl transferease dUTP nick-end labeling (TUNEL) assay and the increased active forms of caspases in the SNpc of postmortem PD tissues including caspase-3, caspase-9, and caspase-8 (Mochizuki et al., 1996; Tompkins et al., 1997; Kingsbury et al., 1998; Tatton et al., 1998). Rotenone is a natural plant compound extracted from certain tropical plant species, which has been extensively utilized as an insecticide and a pesticide (Bové et al., 2005). Low-dose rotenone administration to rats recapitulates most of the mechanisms that are thought to be involved in PD pathogenesis including selective loss of doparminergic neurons and the appearance of Lewy body-like inclusions which are immunopositive for both ubiquitin and α-synuclein, indicating that rotenone is a useful model to explore the pathology and the molecular mechanisms of PD (Cannon et al., 2009). Rotenone triggers the release of proapoptotic factors, such as cytochrome c and Smac/DIABLO from mitochondria into cytoplasm and subsequent activation of caspase-9 and caspase-3 through disrupting mitochondrial membrane potential by binding to complex I to inhibit mitochondrial respiration, which leads to increased reactive oxygen species (ROS) generation (Simon et al., 2000; Lee et al., 2008; Circu and Aw, 2010; Nisticò et al., 2011). Administration of rotenone causes apoptotic cell death in the SNpc of rats by modulating both extrinsic and intrinsic pathway *in vivo* (Ablat et al., 2016). Rotenone also causes morphological characters of apoptosis in both SH-SY5Y and PC12 cells *in vitro* (Lin et al., 2017; Ramkumar et al., 2017). Thus, inhibiting apoptosis resulted from rotenone treatment in SH-SY5Y dopaminergic cells may produce some useful information for the effective treatment of PD in clinical trials.

Proanthocyanidins (PA, C30H26O13, MW 594.52, CAS No. 4852-22-6), also termed condensed tannins, are natural powerful antioxidants widely distributed in many vegetables, fruits, nuts, and seeds, especially in grape seed (Nassiri-Asl and Hosseinzadeh, 2009; Mouradov and Spangenberg, 2014). PA are of great interest in nutrition and medicine because of their various strong biological effects. PA have been demonstrated to have not only anticancer potentials by eliciting apoptosis or impeding cell proliferation but also protective functions by negatively modulating apoptotic signaling pathways (Zhen et al., 2014). It has been reported that PA protect osteoblastic MC3T3-E1 cells against H_2_O_2_-induced apoptosis by ameliorating mitochondrial dysfunction and inhibiting the activation of p53 signaling (Zhang et al., 2014). It has also been suggested that PA exert their protective effect against doxorubicin-induced cardiac injury in rat by reducing the secretion of TNF-α and the activation of caspase-3 (Boghdady, 2013). PA also have neuroprotective effects against various neurotoxicity. For example, PA prevents apoptosis of neurons of hippocampal CA1 area of the mice caused by β-amyloid25-35 toxicity (He et al., 2016). In addition, PA effectively reduce pentylenetetrazole (PTZ)-induced hippocampal dysfunction and improved cognitive decline, in part, by suppressing caspase-3-mediated apoptosis (Zhen et al., 2014). PA extracted from grape seed has also been reported to alleviate rotenone-induced dopaminergic cell death in rat primary mesencephalic cultures (Strathearn et al., 2014). However, little is known about molecular mechanism underlying the potential neuroprotective effect of PA against rotenone-induced cell death in a PD model.

In our study, we aimed to study molecular mechanism underlying the effect of PA on rotenone-induced cell death and in human neuroblastoma SH-SY5Y cells. We show that PA strongly reduced rotenone-induced ROS generation. In addition, PA protected SH-SY5Y cells against rotenone-induced apoptosis. Moreover, we demonstrated that PA antagonized SH-SY5Y cells against rotenone neurotoxicity through suppressing the activation of p38, JNK, and ERK signaling pathways.

## Materials and methods

### Materials

Rotenone was obtained from Sigma-Aldrich Co., LLC (St. Louis, MO, USA), and PA (CAS no. 4852-22-6) was purchased from Yuan Ye (Shanghai, China). Antibodies against β-actin, cleaved caspase-9, cleaved caspase-3, cleaved PARP, phospho-ERK1/2, phospho-p38, p38, phospho-JNK1/2 were purchased from Cell Signaling Technology (Beverly, MA, USA), anti-ERK2 and JNK1 were purchased from Santa Cruz Biotechnology (Santa Cruz, CA, USA). The 4, 6-diamidino-2-phenylindole dihydrochloride (DAPI) was purchased from Sangon (Shanghai, China). Inhibitors SB203580 (p38 MAPK) and SP600125 (JNK), were from Sigma Aldrich. Inhibitor U0126 (MEK) were from Cell Signaling Technology. The One-step TUNEL apoptosis assay kit was purchased from Beyotime (Shanghai, China). Fluorometric Intracellular ROS Kit was purchased from Sigma-Aldrich Co., LLC (St.Louis, MO, USA).

### Cell cultures and drug treatment

Human neuroblastoma cell line SH-SY5Y (kindly supplied by Dr. Evelyne Goillot, Laboratoire d'Immunologie, Centre Leon Berard, France and Eva Feldman, University of Michigan, USA) were cultured in Dulbecco's modified Eagle's medium (DMEM) with 10% fetal bovine serum (FBS) and 1 % penicillin—streptomycin in a humidified incubator at 37°C and at 5% carbon dioxide concentration. Rotenone and PA were dissolved with dimethylsulphoxide (DMSO). The final concentration of DMSO was <0.2% when reagents was added to the experimental cells.

### Cell viability assay

The viability of cells was assessed with MTT assay. In brief, 1 × 10^4^ cells were plated into 96-well plates and incubated overnight. Cells were then washed with fresh medium without serum to remove cell debris and treated with different reagents. Before treating cells with rotenone, the cells were preincubated with PA, SB203580 (p38 MAPK), SP600125 (JNK) or U0126 for 1 h, respectively. When exposed to different treatments for the indicated times duration, cells were treated with 1 mg/mL MTT for 4 h at 37°C and then with DMSO overnight. Absorbance was determined at 490 nm with SpectraMax Plus absorbance microplate reader (Molecular Devices, USA) and then normalized by scaling to the mean of control cells (defined as 100%). Each assay was performed in triplicate and repeated three times.

### Determination of ROS generation

Intracellular ROS production was examined by using Fluorometric Intracellular ROS Kit according to the manufacturer's manual. Briefly, 1 × 10^4^ cells were seeded into 96-well plates. Following treatment with master reaction mix for 1 h, ROS was induced by incubating cells with rotenone or rotenone plus PA (6 μg, 12 μg) for 4 h. The cells were examined by fluorescence microscope (Leica, Wetzlar, Germany). In the meanwhile, the fluorescence readings were determined at 525 nm with molecular devices SpectraMax i3x absorbance microplate reader (Molecular Devices, USA) and then normalized by scaling to the mean of control cells (defined as 100%). Each assay was performed in triplicate and repeated three times.

### Western blot

After different treatments, cells in 6 cm dishes were washed twice with cold PBS (pH 7.4) and then lysed in ice cold lysis buffer (20 mmol/L Tris pH 7.4, 150 mmol/L NaCl, 1 mmol/L Na2EDTA, 1 mmol/L EGTA, 5 mmol/L NaF, 1% Triton X-100, 2.5 mmol/L sodium pyrophosphate, 1 mmol/L β-glycerolphosphate, 1 mmol/L Na3VO4, 1mg/L leupeptin, and 0.5% Na-deoxycholate) and centrifuged at 15,000 g for 30 min at 4°C. The amounts of proteins in the supernatant were determined with a Protein Assay Kit II (BioRad, Hercules, USA). Equal amounts of protein were separated by SDS-PAGE and transferred to a polyvinylidene difluoride membrane (Millipore, Billerica, CA, USA). After blocking with 5% nonfat milk in Tris–HCl buffer (0.1% Tween-20 in 20 mM Tris–HCl, pH 7.6) for 1 h, the membranes were incubated with different primary antibodies (β-actin, cleaved caspase-9, cleaved caspase-3, cleaved PARP, phospho-ERK1/2, ERK2, phospho-p38, p38, phospho-JNK1/2 and JNK1) at 4°C overnight. After washing with Tris-buffered saline/Tween 20 (TBST) for three times (5 min each), the membranes were further incubated with secondary antibodies labeled with horse radish peroxidase (Vazyme Biotech, Nanjing, China) and visualized with Pierce's West Pico Chemiliuminescence substrate. The immunoreactive bands were analyzed by a luminescent image analyzer (Amersham Imager 600, GE Healthcare). The density of immunoreactive protein band was determined by the software ImageJ 1.50 (NIH).

### Tunel staining

Detection of DNA fragments *in situ* was carried out with the one-step TUNEL apoptosis assay kit (Beyotime, Shanghai, China). Briefly, cells were fixed with 4% paraformaldehyde in PBS, permeabilized with 0.1% Triton X-100, and washed with PBS. Cells were then incubated with TUNEL reaction mixture for 1 h at 37°C in the dark. Finally, the cells were washed with PBS for 3 times before examination with fluorescent light microscope (Leica, Wetzlar, Germany).

### Statistical analysis

Data were reported as mean ± SE. Statistical comparisons were determined by *post-hoc* testing using Bonferroni's method. The significance of difference was defined by *P* < 0.05.

## Results

### Effects of PA and rotenone on the viability of SH-SY5Y cells

**Figure 1:**
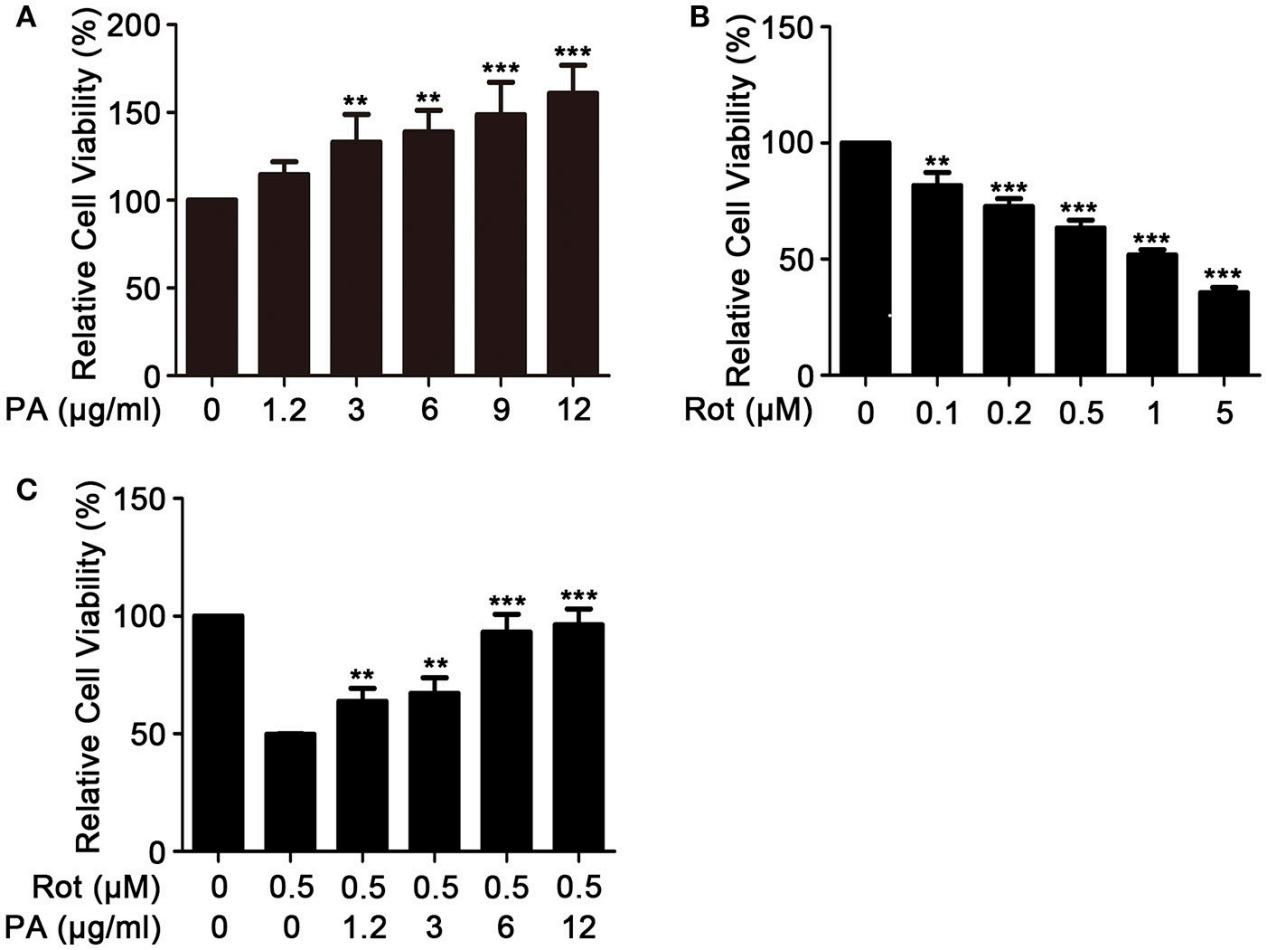
Effects of PA and rotenone on cell viability of SH-SY5Y cells. Cells were incubated with various concentrations of PA **(A)**, Rotenone **(B)** for 24 h, or PA plus Rotenone **(C)** for 36 h. Cell viability was determined by MTT assay. Data are from experiments performed in triplicate and repeated 3 times. ^**^P < 0.01, ^***^P < 0.001, compared with rotenone-treated cells.

We first determined whether PA was toxic to SH-SY5Y cells with MTT assay. Exposure to high concentration of PA significantly increased the viability of SH-SY5Y (Figure 1A). We then evaluated the neurotoxic effect of rotenone on cell survival of SH-SY5Y cells. The result revealed that rotenone greatly reduced cell survival in a dose-dependent manner with a cell survival rate of 63 ± 1.69% in response to 0.5 μM rotenone treatment for 24 h (Figure 1B). Consequently, 0.5 μM rotenone was used in the following experiments. Finally, we assessed the protective effect of PA on rotenone-induced cell death. Before treating cells with rotenone to induce cell death, cells were preincubated with PA for 1 h. As shown in Figure 1C, PA greatly increased the viability of SH-SY5Y cells. The maximum protection occurred at 6 μg of PA and further increasing PA concentration to 12 μg only slightly elevated cell viability, but the elevation was not significant in comparation with the viability at 6 μg of PA (*P* > 0.05).

### Effects of PA on rotenone-induced oxidative stress

**Figure 2:**
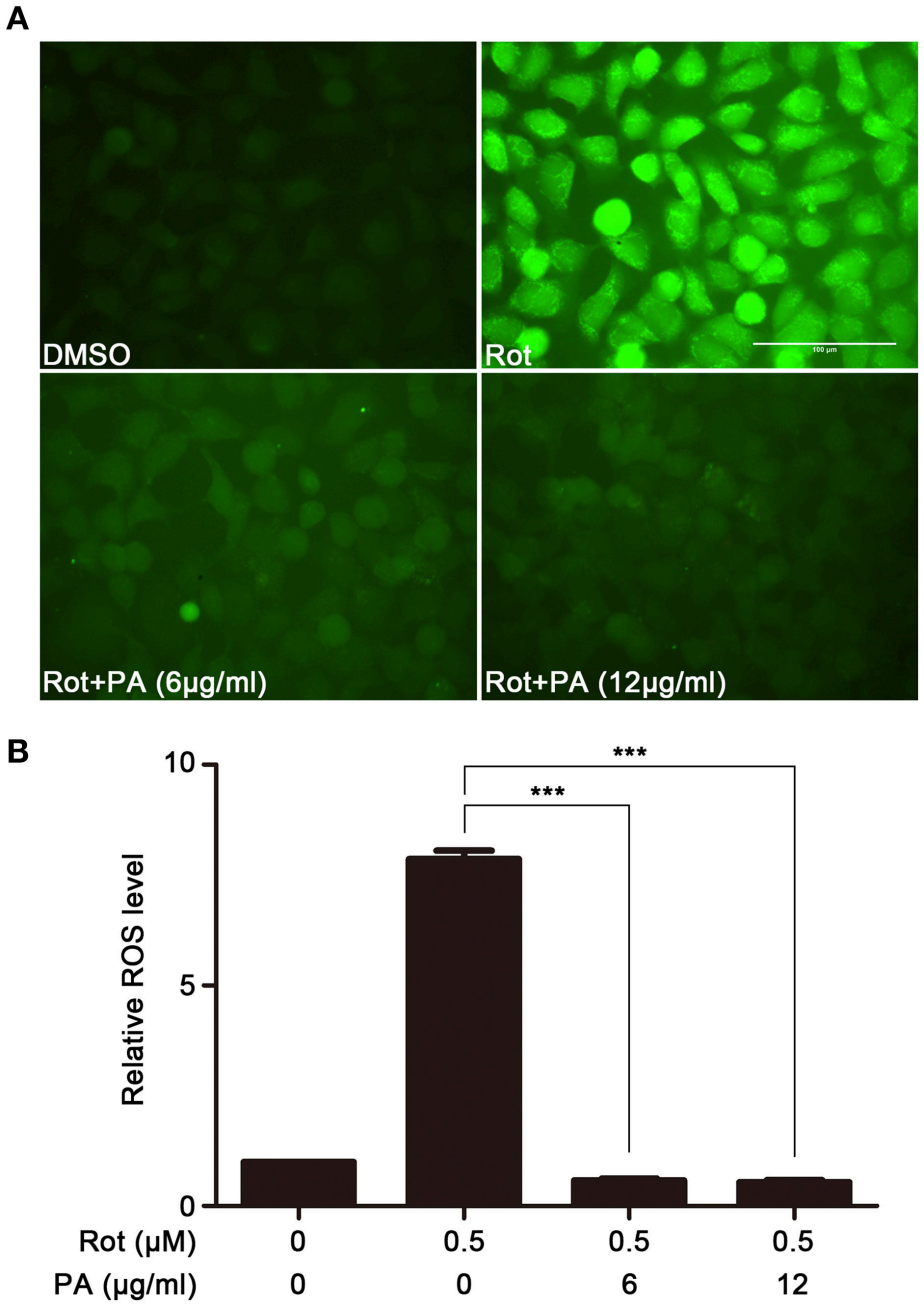
Effects of PA on rotenone-induced ROS generation. **(A)** Representative images showing the preventive effect of PA against rotenone-induced ROS production. Scale bar: 100 μm. **(B)** The fluorescence readings were determined with microplate reader. Data are from experiments performed in triplicate and repeated 3 times. ^***^P < 0.001, compared to control.

To examine the effect of PA on rotenone-induced production of intracellular ROS in SH-SY5Y cells, ROS amount in SH-SY5Y cells was determined using Fluorometric Intracellular ROS Kit. The generation of intracellular ROS significantly enhanced after incubation with rotenone. In contrast, preincubation with PA (6 μg, 12 μg) markedly inhibited rotenone-induced ROS production (Figures 2A,B).

### PA ameliorated rotenone-induced apoptosis

**Figure 3:**
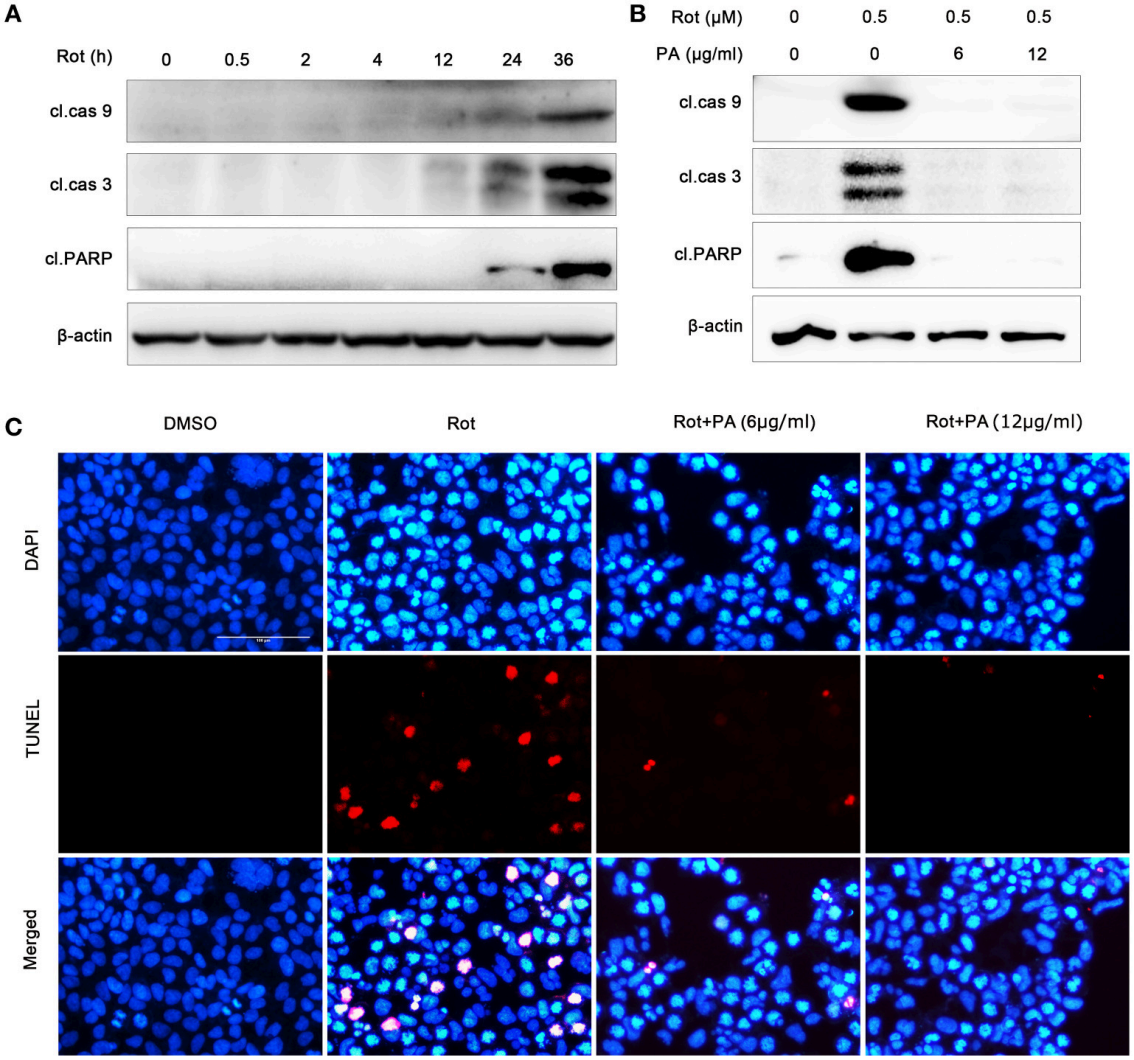
PA protect human neuroblastoma SH-SY5Y cells against rotenone-induced apoptotic cell death. **(A)** Rotenone induced activation of caspase-9, caspase-3 and cleavage of PARP in 24 and 36 h. Samples were assessed by western blot with antibody against the cleaved caspase-9, caspase-3 and PARP, and β-actin was used as loading control. **(B)** PA reduced the activation of caspase-9, caspase-3 and cleavage of PARP induced by rotenone. Cells were treated with rotenone or rotenone plus PA (6 or 12 μg) for 36 h. Samples were assessed by western blot with antibody against the cleaved caspase-9, caspase-3 and PARP, and β-actin was used as loading control. **(C)** SH-SY5Y cells were treated with rotenone or rotenone plus PA (6 or 12 μg) for 36 h, and then apoptosis was detected by TUNEL (red) staining, nuclei were stained by DAPI (blue). Scale bar: 100 μm. Results are representative of at least three experiments.

It had been reported that apoptosis was involved in rotenone-induced cell death (Lin and Beal, 2006). So we tested whether PA could antagonize rotenone-induced apoptosis in SH-SY5Y cells. As shown in Figure 3, biological markers of cell apoptosis, cleavage of caspase-9, caspase-3 and poly (ADP-ribose) polymerase (PARP) which was a substrate of caspases (Virág et al., 2013) were greatly induced after exposure to 0.5 μM rotenone for 24 and 36 h (Figure 3A), whereas PA greatly inhibited rotenone-caused cleavage of caspase-9, caspase-3 and PARP (Figure 3B). To further assess the neuroprotective effects of PA against rotenone neurotoxicity in SH-SY5Y cells, we performed TUNEL staining assay. TUNEL analysis showed that rotenone induced a significant increase in the number of apoptotic nuclei, whereas PA (6 or 12 μg) markedly reduced the number of apoptotic nuclei in SH-SH5Y cells (Figure 3C). Taken together, these results indicate that PA could confer neuroprotection in SH-SY5Y cells against rotenone-induced apoptotic cell death.

### PA suppressed the activation of p38, JNK, and ERK pathways induced by rotenone

**Figure 4:**
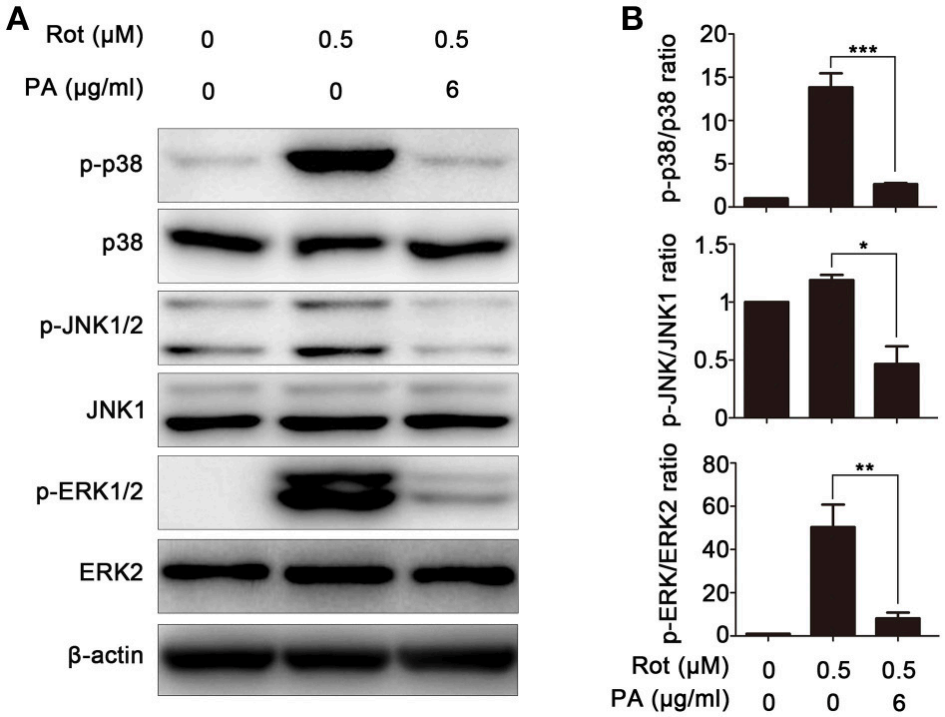
Effects of PA on rotenone-induced ERK, p38, JNK, and ERK phosphorylation in SH-SY5Y cells. **(A)** Cells were pretreated with various doses (6 μg) of PA for 4 h before rotenone (0.5 μM) stimulation for 1 h. Samples were assessed by western blot with antibody against p-p38, p-ERK1/2, and p-JNK. A non-phosphorylated form of each targeted protein was used as loading control. β-Actin was used as an internal control. Results are representative of at least three experiments. **(B)** The levels of phosphorylated ERK, p38, and JNK were quantified by densitometry, ^*^p < 0.05, ^**^p < 0.01, ^***^p < 0.001, vs. control group.

To determine the molecular mechanism underlying PA-mediated neuroprotective effect against rotenone-induced cell death, multiple potential signaling pathways which have been demonstrated to be engaged in rotenone-induced apoptosis were screened. As revealed by western blot, rotenone incubation leads to enhanced activation of p38, JNK, and ERK, whereas PA significantly down-regulated the activation of p38, JNK, and ERK signaling pathways, indicating that PA may inhibit rotenone-induced apoptosis via ERK, p38, and JNK signaling pathways (Figures 4A,B).

### PA-mediated neuroprotection is dependent on the inhibition of p38 signaling

**Figure 5:**
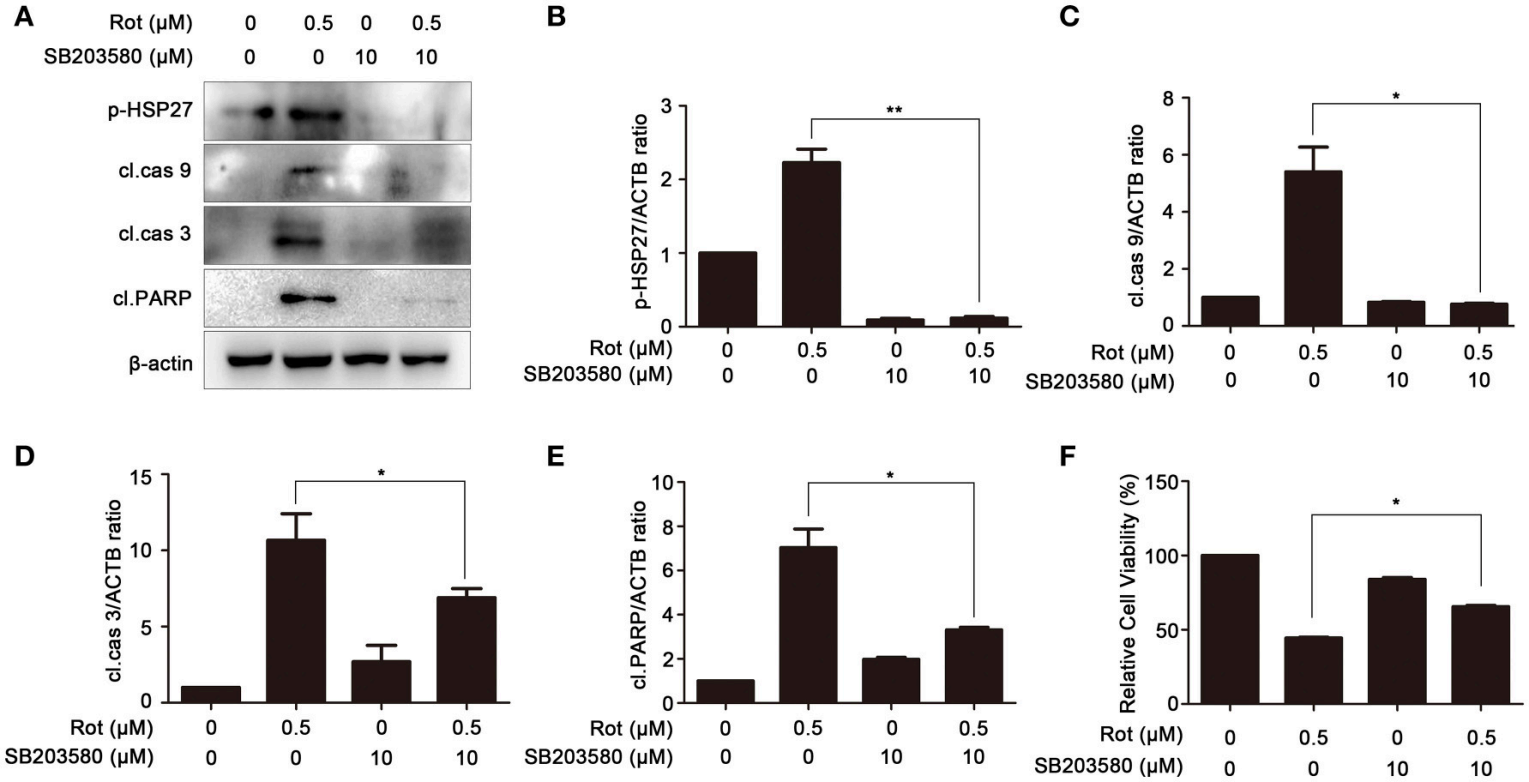
Inhibition of p38 signaling prevents rotenone-induced apoptosis. **(A)** The SH-SY5Y cells were pretreated with 10 μM SB203580 (SB) for 1 h, and then treated with rotenone for 36 h, cleaved caspase-9, cleaved caspase-3, cleaved PARP and p-HSP27 were detected by western blot, β-actin was used as a loading control. Results are representative of at least three experiments. The levels of p-HSP27 **(B)**, cleaved caspase-9 **(C)**, cleaved caspase-3 **(D)**, cleaved PARP **(E)** were quantified by densitometry. ^*^p < 0.05, ^**^p < 0.01, compared with rotenone-treated group. **(F)** In the presence or absence of SB, cells were treated with rotenone for 36 h, cell viability was assessed by MTT assay. Data are expressed as percent of values in untreated control. ^*^P < 0.05, compared with rotenone-treated cells.

To determine the involvement of p38 MAPK signaling pathways in PA-mediated cell protection, SH-SY5Y cells were incubated with rotenone with or without SB203580, a specific inhibitor of p38 signaling pathway. To determine the efficiency of SB203580 in inhibiting p38 signaling, we first examined the phosphorylation of heat shock protein 27 (p-HSP27), a downstream effector of p38, by western blot. As shown in Figures 5A,B the phosphorylation of p-HSP27 induced by rotenone was greatly reduced by SB203580, suggesting that activation of p38 signaling pathway caused by rotenone is effectively inhibited by SB203580. We then determine the expression of the apoptotic markers, cleaved caspase-9, cleaved caspase-3, and cleaved PARP. In comparison with cells incubated with rotenone, cleaved caspase-9, cleaved caspase-3, and cleaved PARP was strongly downregulated by SB203580 (Figures 5A,C–E). Cell viability of cells pretreated with SB203580 was also markedly increased from 44 ± 0.71 to 66 ± 0.90% in comparison with rotenone-treated cells (Figure 5F). These results suggest that PA may prevent rotenone-induced apoptosis by prohibiting p38 signaling.

### PA-mediated neuroprotection is dependent on the inhibition of JNK signaling

**Figure 6:**
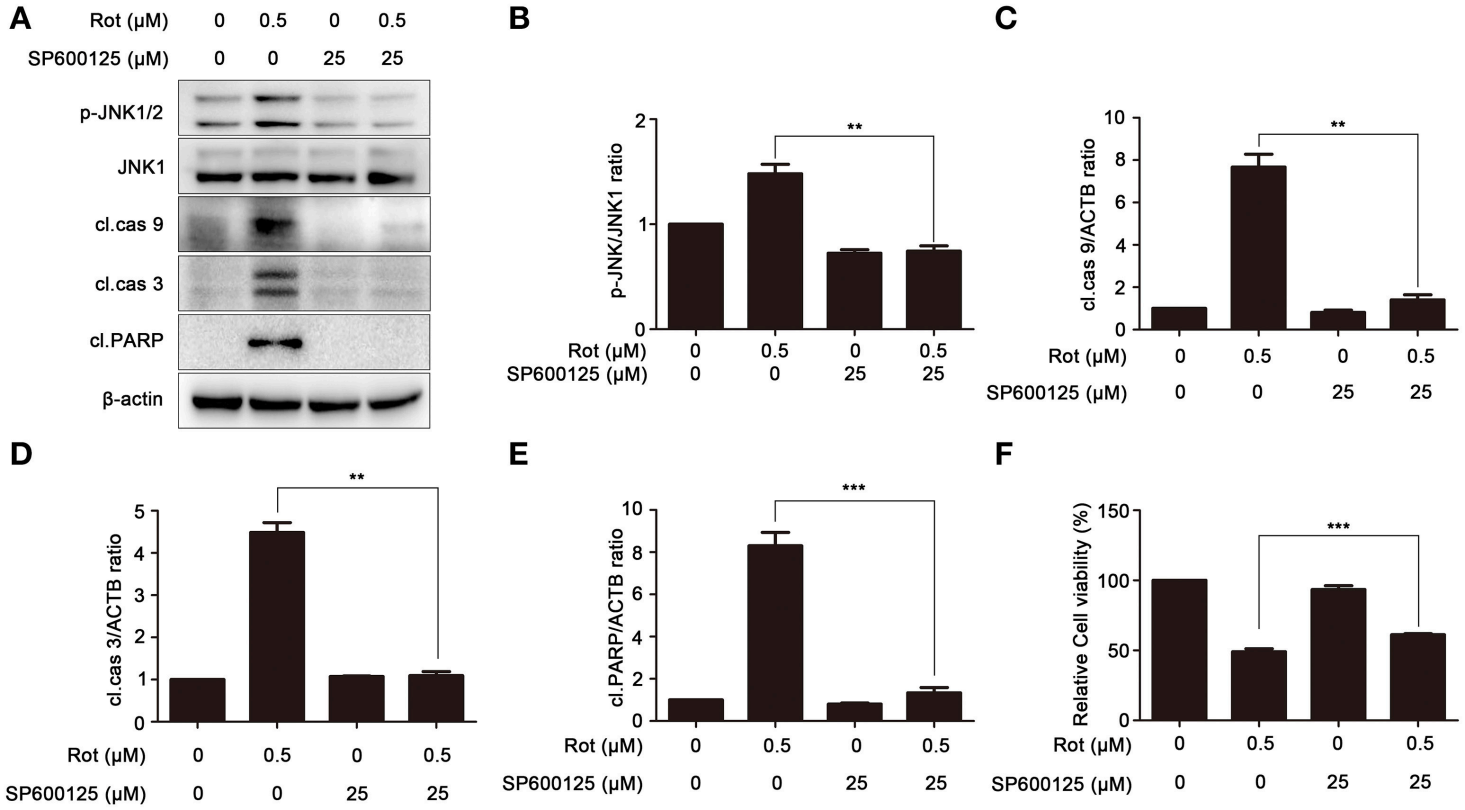
Inhibition of JNK signaling prevents rotenone-induced apoptosis. **(A)** The SH-SY5Y cells were pretreated with 25 μM SP600125 (SP) for 1 h, and then treated with rotenone for 36 h, cleaved PARP, p-JNK and JNK1 were detected by western blot, JNK1 was used as loading control, β-Actin was used as an internal control. Results are representative of at least three experiments. The levels of p-JNK **(B)**, cleaved caspase-9 **(C)**, cleaved caspase-3 **(D)**, cleaved PARP **(E)** were quantified by densitometry. ^**^p < 0.01, ^***^p < 0.001 vs. rotenone-treated group. **(F)** In the presence or absence of SP, cells were treated with rotenone for 36 h, cell viability was assessed by MTT assay. Data are expressed as percent of values in untreated control cultures. ^***^P < 0.001, compared with rotenone-treated cells.

To elucidate the contribution of JNK signaling in PA-mediated cell protection, SH-SY5Y cells were treated with rotenone in the presence or absence of SP600125, a specific inhibitor of JNK signaling. We tested the effect of SP600125 on phosphorylation of JNK induced by rotenone treatment with western blot. Compared with cells treated by rotenone, phosphorylation of JNK was significantly decreased by SP600125, indicating that rotenone-induced activation of JNK signaling is effectively downregulated by SP600125 (Figures 6 A,B). The expression of apoptotic marker cleaved caspase-9, cleaved caspase-3, and cleaved PARP was also assessed by western blot. As shown in Figures 6A,C–E, cleaved caspase-9, cleaved caspase-3, and cleaved PARP was strongly reduced by SP600125 compared with cells treated with rotenone. To further examine the contribution of JNK signaling in PA-mediated cell survival, we pretreated SH-SY5Y cells with SP600125 for 1 h before rotenone treatments. Cell viability was determined 24 h after incubation. In cells induced with rotenone, the cell viability was about 49 ± 2.07% of the control, whereas SP600125 markedly enhanced the survival of SH-SY5Y cells to 61 ± 0.74% (Figure 6F). These results suggested that PA prevent rotenone-induced apoptosis by blocking JNK signaling.

### PA-mediated neuroprotection is dependent on the inhibition of ERK signaling

**Figure 7:**
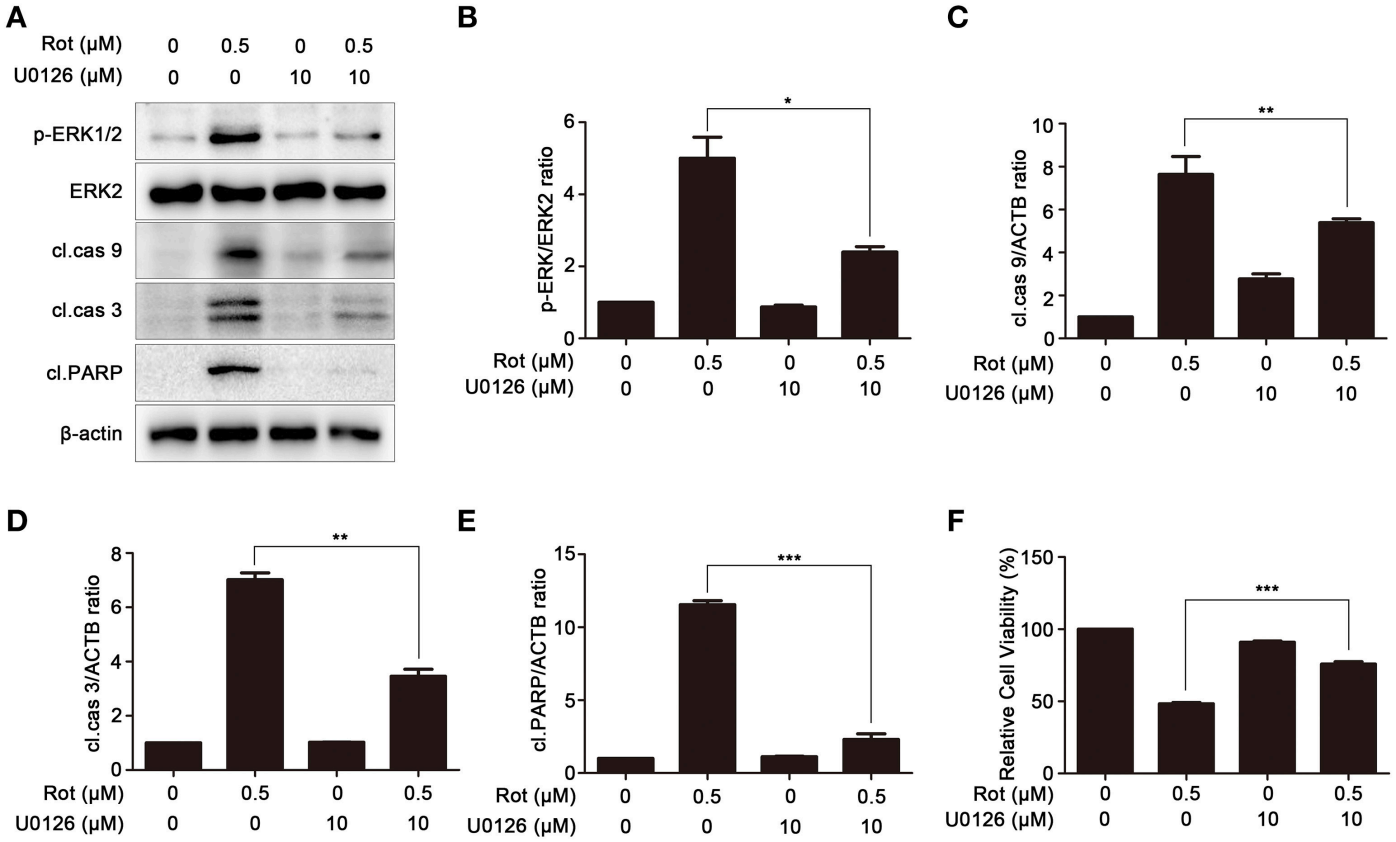
Inhibition of ERK signaling prevents rotenone-induced apoptosis. **(A)** The SH-SY5Y cells were pretreated with U0126 (10 μM) for 1 h, and then treated with rotenone for 36 h, cleaved PARP, p-ERK, ERK2 were detected by western blot, ERK2 was used as loading control, β-Actin was used as an internal control. Results are representative of at least three experiments. The levels of p-ERK **(B)**, cleaved caspase-9 **(C)**, cleaved caspase-3 **(D)**, cleaved PARP **(E)** were quantified by densitometry. ^*^p < 0.05, ^**^p < 0.01, ^***^p < 0.001 vs. rotenone-treated group. **(F)** In the presence or absence of U0126, cells were treated with rotenone for 36 h, cell viability was assessed by MTT assay. Data are expressed as percent of values in untreated control cultures. ^***^P < 0.001, compared with rotenone-treated cells.

U0126, an ERK signaling inhibitor, was employed to confirm the role of ERK in PA-mediated cell protection. SH-SY5Y cells were pretreated with U0126 before incubation with rotenone. We first determined the effect of U0126 on the activation of ERK induced by rotenone with western blot. As shown in Figures 7A,B, rotenone-enhanced activation of ERK was significantly reduced by U0126. In addition, the levels of the apoptotic markers, cleaved caspase-9, cleaved caspase-3, and cleaved PARP were greatly impaired by U0126 in comparison to cells treated with rotenone alone (Figures 7A,C–E). Consistently, cell viability was markedly increased from 48 ± 0.88 to 76 ± 1.65% after pretreatment with U0126 (Figure 7F). These data indicate that PA prevent rotenone-induced apoptosis by inhibiting ERK signaling.

## Discussion

PA are highly effective natural antioxidants that are widely available in a variety of plants. PA possess a broad spectrum of potent properties, such as antioxidant, anti-inflammatory, antiviral and anti-carcinogenic activities (Ye et al., 1999; Fine, 2000; Park et al., 2012; Ding et al., 2013). PA also have neuroprotective effect against free radical-induced diseases, such as PD (Moreira et al., 2010). In the present study, we utilized human neuroblastoma SH-SY5Y cells as an *in vitro* PD cell model to study the effect of PA on rotenone-induced oxidative stress and apoptosis. We for the first time reported that PA effectively reduced the generation of ROS and apoptotic cell death induced by rotenone. Further study revealed that incubation of SH-SY5Y cells with inhibitors of p38, JNK, and ERK signaling recapitulates the neuroprotective effect of PA. Our results may provide a new insight on PA as a potent agent in the effective treatment of PD.

Rotenone recapitulates the pathological characters of PD in both *in vitro* and *in vivo* models by increasing the generation of oxidative stress which is involved in degeneration of dopaminergic neurons and implicated in the initiation and progression of PD patients (Lin and Beal, 2006). Therefore, intervention in rotenone-induced oxidative stress has been proposed to play an important function in survival of DA neurons and may provide potential therapeutic benefit for PD (Mao et al., 1999; Junn and Mouradian, 2001). Our data suggested that rotenone treatment can induce the production of intracellular ROS in SH-SY5Y dopaminergic cells, whereas PA treatment significantly decreased ROS production in rotenone-treated cells, which might be due to the potent ability of PA to scavenge hydroxyl radicals or to upregulate the activity of antioxidant enzymes, such as glutathione peroxidase (GSH-Px) and superoxide dismutase (SOD) (Cai et al., 2016).

Stress-activated signaling pathways, including p38 and JNK, are crucial to apoptotic cell death in neurons. p38 signaling often plays a pro-apoptotic role in response to various insults, although in some cases p38 activation functions as a compensatory response or an anti-apoptotic mechanism (Kawasaki et al., 1997; Kummer et al., 1997; Schwenger et al., 1997; Mao et al., 1999; Ghatan et al., 2000; Caughlan et al., 2004). For example, apoptosis induced by dopamine, rotenone, or trophic deprivation in SH-SY5Y cells is mediated by p38 activation (Kummer et al., 1997; Junn and Mouradian, 2001; Newhouse et al., 2004). Consistently, our results revealed that PA-mediated cell survival is carried out by suppression of p38 activation. This conclusion is supported by two evidences. First, rotenone-induced p38 activation was inhibited by PA. Second, SB203580, a specific inhibitor of p38, reproduced the protective effect of PA by increasing rotenone-reduced cell survival and decreasing the expression of cleaved PARP, an apoptotic marker.

JNK is another stress-activated MAP kinase that has been engaged in regulating many forms of neuronal apoptotic cell death (Xia et al., 1995; Davis, 2000; Putcha et al., 2003). Here, we demonstrated that JNK is activated by rotenone and this activation is blocked by PA. Moreover, JNK inhibitor SP600125 protects SH-SY5Y cells against apoptosis induced by rotenone, providing a solid evidence that JNK signaling plays a pro-apoptotic function in rotenone-mediated cell death. Besides rotenone, JNK is also involved in the neurodegeneration of other PD models, such as MPTP, 6-hydroxydopamine, and dopamine, indicating that JNK is involved in neurotoxin-induced neurodegenerative processes in PD pathogenesis (Wang et al., 2004; Crocker et al., 2011; Zhang et al., 2016). Thus, agents that target JNK signaling may slow down the progression of PD by maintaining dopamine producing neurons that have not yet been lost in the SNpc of PD patients. Our data suggest that PA may be serving as such agents based on its ability to protect SH-SY5Y cells from rotenone neurotoxicity by inhibiting JNK signaling.

MAPK/ERK signaling can be either pro- or anti-apoptotic depending on types of cells and insults (Wang et al., 2010). It has been reported that ERK protects neurons from apoptotic cell death from drugs that induce DNA damage, deprivation of tropic factors or ischemia (Bonni et al., 1999; Jin et al., 2002; Gozdz et al., 2003). ERK has also been shown to facilitate neural cell death induced by glutamate or okadaic acid (Rundén et al., 1998; Satoh et al., 2000; Stanciu et al., 2000). In the present study, we found that blockage of ERK activation induced by rotenone with U0126 reproduced the protective effect of PA. These results indicate that MAPK/ERK is pro-apoptotic in rotenone-induced cell death and PA may exert it neuroprotective function by inhibiting ERK signaling.

Our results indirectly point out that PA's neuroprotective effects are due to the suppression of p38, JNK, and ERK. This conclusion is based on two evidences. First, PA inhibits rotenone-induced activation of p38, JNK, and ERK. Second, inhibitors to p38, JNK, and ERK can partially suppress rotenone-induced apoptosis in SH-SY5Y cells. In comparison with single inhibitor, PA can antagonize all three signaling pathways at the same time. It may explain why individual inhibitor to p38, JNK, and ERK only partially suppresses rotenone-induced apoptosis, while PA possesses robust protective effects on PA neurotoxicity.

In summary, PA reduces ROS generation and protects against apoptotic cell death induced by rotenone in SH-SY5Y cells via suppressing p38, JNK, and ERK signaling pathways. However, it remains unclear how PA modulates these signaling pathways and whether there is any crosstalk between these signaling pathways. Further study is needed to clarify these issues in the future.

## Conclusion

In the current study, we demonstrated that PA effectively decreases ROS production and antagonizes rotenone-induced apoptosis in SH-SY5Y cells. Moreover, we showed that PA-promoted cell survival is mediated by suppressing activation of p38, JNK, and ERK signaling. Our data may support the possible efficacy of PA in PD treatment.

## Author contributions

LW conceived and designed the experiments. JM, SS-G, H-JY, MW, and B-FC performed the experiments. B-FC, Z-WF, and LW analyzed the data. LW wrote the paper.

### Conflict of interest statement

The authors declare that the research was conducted in the absence of any commercial or financial relationships that could be construed as a potential conflict of interest.
